# Mass distribution campaign of long-lasting insecticidal nets (LLINs) during the COVID-19 pandemic in Uganda: lessons learned

**DOI:** 10.1186/s12936-023-04753-6

**Published:** 2023-10-16

**Authors:** Herbert Bush Aguma, Medard Rukaari, Rukia Nakamatte, Pamela Achii, Joel Tutu Miti, Solomon Muhumuza, Mariam Nabukenya, Jimmy Opigo, Martin Lukwago

**Affiliations:** 1 National Malaria Control Division, Ministry of Health, Kampala, Uganda; 2 Department of Pharmacy, Makerere University, Kampala, Uganda; 3 Department of Planning, Finance & Policy, Ministry of Health, Kampala, Uganda

**Keywords:** COVID-19, LLIN Campaign, Lessons Learnt

## Abstract

**Background:**

Uganda implemented its third mass campaign to distribute long-lasting insecticidal nets (LLINs) in 2020 during the COVID-19 pandemic. This context necessitated modification of implementation guidelines. The mass campaign's objective was to ensure that at least 85% of the targeted population had access to LLINs.

**Methods:**

Revised implementation guidelines were followed while conducting the LLIN distribution campaign. Lessons learned were captured from documented activities and reports.

**Results:**

A total of 27,789,044 mosquito nets were distributed in 11,287,392 households, with an average of 5.1 persons per household. Household coverage of the LLIN distribution was 94.1%. The 2020/2021 campaign design was modified to follow COVID-19 Standard Operating Procedures (SOPs). These included using Personal Protective Equipment (PPE), e-platforms for training and briefing meetings, electronic data management systems and door-to-door household registration and distribution of LLINs.

**Conclusions:**

Campaign modifications due to the COVID-19 pandemic were effective in implementing mass distribution of LLINs despite the disruptions and restrictions. The campaign’s net coverage far exceeded its objective. Electronic data management was critical in monitoring and reporting distribution activities.

## Background

Malaria remains a globally endemic disease, with an estimated 241 million cases and 627,000 deaths in 2020. There was an increase in malaria cases by 6.2% from 2019, with most of this increase accounted for by the countries in the World Health Organization (WHO) Africa region. The increase is attributed to the disruption of health services during the COVID-19 pandemic. Uganda accounted for 5% of the malaria cases and deaths globally, making it a country with the third-highest number of cases and deaths [1]. Uganda is one of the eleven high burden high impact (HBHI) countries where malaria cases did not change significantly between 2018 and 2019 [2]. However, compared to the previous year, Uganda saw a significant decrease in malaria cases in 2018 [1]. Despite a decrease in malaria cases in 2018, Uganda's contribution to global malaria cases has remained at 5% [1–5].

When the COVID-19 pandemic began, it was predicted that Africa's gains in malaria control would be lost due to disruptions in malaria management interventions and overburdened health systems. Statistical modelling predicted that malaria cases and deaths would double [2, 3]. Societal restrictions intended to reduce the spread of COVID-19 were expected to impede access to healthcare services, disrupt service delivery, and postpone malaria prevention activities such as LLIN and IRS campaigns [4].

On March 11, 2020, the WHO declared COVID-19 a global pandemic. Uganda had no case of COVID-19 until March 21, 2020. The Government of Uganda immediately instituted strict restrictions in response to the declaration of the pandemic and the arrival of the first COVID-19 case. The restrictions included suspension of public gatherings, social distancing in public places, lockdown and quarantine, border closures, face mask mandate, school closures, a ban on public transport, and curfews. The lockdown measures negatively impacted the healthcare system and access [5, 6]. Non-COVID-19 patients curtailed their attendance at healthcare facilities due to limited public transportation and the fear of catching COVID-19 [7–9]. Healthcare staff attendance was limited, citing reasons like movement restrictions, lack of accommodation at workstations, and shortage of PPE [10]. Health facilities experienced shortages of essential medicines and health supplies [11].

Despite the detrimental consequences of COVID-19-related restrictions on Ugandan health care services, a study conducted in rural Ugandan health facilities found that the first year of the pandemic had no significant influence on malaria disease burden and case management parameters. Furthermore, malaria prevention initiatives such as the mass LLIN distribution campaign and IRS were successfully implemented [12].

The WHO recommends universal coverage with insecticide-treated nets (ITNs) for all populations at risk of malaria in endemic areas [13]. ITNs have been a cost-effective and most-used vector control method in reducing the number of malaria cases in endemic areas, accounting for a significant reduction of malaria cases since 2000 [14]. Moreover, access to mosquito nets in households has been shown to increase after mass distribution campaigns for LLINs [15].

The Government of Uganda adopted the policy of mass distribution of ITNs as one of the significant interventions for malaria prevention. Three mass campaigns have been implemented in 2013–2014, 2017–2018, and 2020–2021 [15, 16]. The first distribution of LLINs in 2010 targeted only pregnant women and children. Following the targeted LLIN distribution in 2010, three mass campaigns targeting the whole country have been implemented [15]. Prior to the emergence of COVID-19, mass distribution campaigns were designed to include the following key steps: planning and coordination, quantification of the number of LLINs required, procurement of LLINs, social mobilization and sensitization, training of district supervisors, health workers, and volunteers, distribution strategy utilizing distribution points, manual data collection, and monitoring [15].

The recently concluded mass ITN distribution occurred from July 2020 to March 2021. It was implemented during the COVID-19 pandemic and was characterized with electronic data for the first time to manage beneficiary data collection and household delivery of ITNs [17]. COVID-19-related disruptions necessitated modification of the initial campaign design to avoid postponement of LLINs distribution. This article describes the implementation of the mass campaign during the COVID-19 pandemic and the lessons learned.

## Methods

### Context

Uganda has a population of 43.7 million. The country has 146 districts, 1488 sub-counties, and 58,197 villages. Over 90% of the country has stable, perennial transmission, with transmission peaks related to the two annual rainy seasons.

The outbreak of the COVID-19 pandemic was reported in Uganda on March 21, 2020, when the first patient landed at Entebbe International Airport [6]. The country was after that placed under several lockdown measures. COVID-19-related restrictions slightly disrupted the ITN campaign's commencement, slated to begin in March 2020. However, the campaign began in July 2020 and ended in March 2021. The objective of the LLIN campaign was to ensure that at least 85% of the targeted population had access to LLINs [18].

Uganda undertook a series of COVID-19-related lockdowns in 2020 following the declaration of the pandemic by WHO. The initial national lockdown began on March 30, 2020, and lasted for 42 days until May 5 2020. Subsequent lockdowns eased restrictions, allowing essential services and workers to travel and work [19].

The contexts of the previous two mass campaigns are comparable [18]. They both aimed to achieve universal coverage while preventing malaria, a significant public health concern in Uganda. Both campaigns occurred when no pandemic outbreak occurred, which would have necessitated restrictions. The first campaign in 2013/2014 aimed to increase LLIN ownership, particularly among vulnerable groups such as pregnant women and children under five. Before the campaign, household ownership of LLINs was low (59%) [20]. The second campaign of 2017/2018 aimed to build on the previous campaign's gains and lessons learned to reach hard-to-reach areas and maintain household coverage. Both campaigns followed implementation guidelines that remained unchanged except for adding best practices to lessons learnt [21].

### Implementation guidelines of the LLIN mass campaign

The guidelines for implementation were first introduced in 2010 and have since undergone slight changes premised on lessons learned from previous mass campaigns [21, 22]. The guidelines provide detailed guidance on planning, implementing, monitoring, and evaluating a mass campaign. They also define all stakeholders' and partners' roles and responsibilities. The Ministry of Health (MOH), WHO, Alliance for Malaria Prevention (AMP), President’s Malaria Initiative (PMI), and Global Fund to fight AIDS, Tuberculosis and Malaria (GFATM) collaborated to develop the 2020 guidelines derived from AMP guidance. Coordination and oversight, procurement of LLINs and microplanning, campaign personnel selection and training, household registration, LLIN allocation and distribution, monitoring and evaluation, risk management plan, and COVID-19 adaptations were the critical elements of the guidelines.

The guidelines outline the National Malaria Control Division (NMCD)'s role in directing the implementation process and coordinating all stakeholders. Campaign coordination and supervision structures exist at the central, regional, district, sub-county, parish, and village levels. The NCC takes the lead at the central and regional levels, supported by four technical subcommittees: operations, logistics, M&E, and SBC. At the district, sub-county, parish, and village levels, there is a district task force, a sub-county task force, a parish chief, and an LC I, respectively.

The Uganda Bureau of Statistics (UBOS) 2020 population projection was used to perform macro quantification of LLINs. The WHO recommendation of one LLIN per two people was followed, with a 10% buffer included. LLINs were obtained through the Global Fund and delivered to National Medical Stores (NMS), responsible for warehousing and transporting them to sub-county stores. Third-party logistics companies were used in prior campaigns to warehouse and distribute LLINs. The macro plan and macro budget were translated into detailed operation plans and budgets for each district in the country using microplanning guidelines.

The campaign's human resources included a national secretariat, microplanning supervisors, and district supervisors at the district level. Cascaded training began with training trainers at the national level and cascaded to the district, sub-county, and parish levels utilizing training manuals.

Household registration at the village level was preceded by household mobilization to inform them about LLIN distribution and to disseminate messages about malaria and its prevention using LLINs. These messages were specified in the SBC plan. A team of two (Village Health Team) VHTs, two (Data Entry Clerks) DECs, two Local Guards, and the (Local Council) LC I chair conducted household registration and LLIN distribution electronically using the EDMS app. Registration of households and distribution of LLINs were done using the door-to-door strategy [17].

### Campaign implementation design

Like prior ones, the campaign was designed to be district-led, with decentralized structures leading in planning and distributing nets at the household level. The National Coordination Committee (NCC), chaired by the Permanent Secretary of the Ministry of Health, oversaw the campaign. It was supported by four technical subcommittees: logistics, social and behaviour change communication (SBCC), operations, and monitoring and evaluation (M & E). Technical people led these in the National Malaria Control Division of the Ministry of Health, and membership was drawn from the NCC. Unlike previous campaigns led by a lead agency [23], the 2020/2021 campaign was institutionalized within the MOH. A procurement and financial management agency, as well as a fiduciary assurance agency, assisted the MOH.

### Campaign implementation process

#### Macroplanning

The ITN need was quantified using the Uganda Bureau of Statistics (UBOS) projected population figures. Types of ITNs deployed in various locations were decided based on the insecticide resistance patterns. Three (3) types of nets were ordered: Standard (Non-PBO, PBO) and dual-active. Over 95% of the ITNs procured were polyester due to the limited acceptance of polyethylene nets in the previous campaign of 2017/2018.

#### Microplanning

A technical team was trained and provided with a microplanning tool developed consultatively with malaria partners in Uganda and the Alliance for Malaria Prevention (AMP). For each administrative unit, the Microsoft Excel tool was meant to collect qualitative and quantitative data on operations, SBC, M&E, and logistics. Using this tool, data was collected from districts. Data collection teams went up to sub-county and village levels. Previous campaigns collected microplanning data by visiting only up to the sub-county level. Data on population and administrative units were aggregated for each district from the lowest level, i.e. a village and compared with UBOS data. Where there was a variance above 10%, the data was validated using other sources like the Uganda Electoral Commission. The data was validated and produced in district micro plans stipulating the net and non-net deliveries to each sub-county. The micro plan also had a district budget broken down into sub-county budgets based on the administrative units in each district. Previous campaigns collected microplanning data only up to the sub-county level. Data on population and administrative units were aggregated from the lowest level, a village, for each district and compared to UBOS data. Where there was more than a 10% difference, the data was double-checked using other sources, such as the Uganda Electoral Commission and previous campaign statistics. Following data analysis, summarized micro plans were distributed to districts via regional advocacy meetings through printed booklets.

#### Procurement and delivery of LLINs

The ITNs were obtained with funding from GF and AMF. Quantities of nets required for each wave were quantified. Orders were made a year earlier to allow for the manufacture and shipment of orders. Delivery was also staggered due to warehouse storage constraints. A total of 28,805,800 nets were procured. They were warehoused at the National Medical Stores (NMS). The NMS was also responsible for the delivery of nets up to sub-county stores. As a result, the pre-existing distribution mechanism for pharmaceuticals and health supplies was used. Microplanning data collected from districts was used to decide the quantities allocated per sub-county in each district. Logistics tools, namely stock cards, waybills and tally sheets, were used for tracking and accountability for LLINs and other non-LLIN commodities across the supply chain. Delivery of nets to each village was based on household registration data per day. These nets were collected from pre-positioning centres located at the parish level. Excess nets were redistributed or committed to reverse logistics where possible.

The strict requirement of testing transporters at the border crossing point through which the LLINs entered the nation caused a delay in the delivery of LLINs at the beginning of the campaign. LLIN shipments were also delayed because of COVID-19's impact on global supply systems. The last shipment was received in March 2021, as opposed to its anticipated arrival date of June 2020.

#### Training

Training manuals were utilized to guide training. A standard training curriculum was created with content on all practical aspects of campaign implementation at the district, sub-county, parish, and village levels. A training of trainers that included secretariat staff and district supervisors served as the foundation for training. The training was then cascaded down to sub-county supervisors, who trained people in the district. Four training sessions were held in the district: district and sub-county technical team training, VHTs, DECs, store personnel, LCIs and local guards training, and parish chiefs training. Data collectors needed practical training to use phones and install the electronic data management information system (EDMIS) app.

#### Household registration

Data entry clerks went to each household and collected data from an adult household head. Registration determined the number of nets delivered to the households by the Village Health Teams (VHTs) and Local guards. Each household was limited to receiving a maximum of five LLINs. The maximum number of LLINs given to each household was capped to combat oversupply in instances where household members.

#### Mass distribution

Distribution of LLINs at the household level was done by moving from house to house after registration, except for a few sparsely populated areas. Household members were trained to use the net and where to hang it.

#### Supervision

Supervision was done by sub-county and district supervisors who also collaborated with Parish Chiefs. Supervision was also assisted by district and sub-county task force members.

#### Electronic data management

An electronic data management system was used to monitor and implement all campaign activities. The EDMIS (Electronic Data Management Information System) software was developed to digitize and monitor household data. The software used in the 2017–2018 campaign was upgraded to EDMIS. The EDMIS Android Mobile app and the EDMIS web-based system were the two main components of EDMIS.

**Fig. 1:**
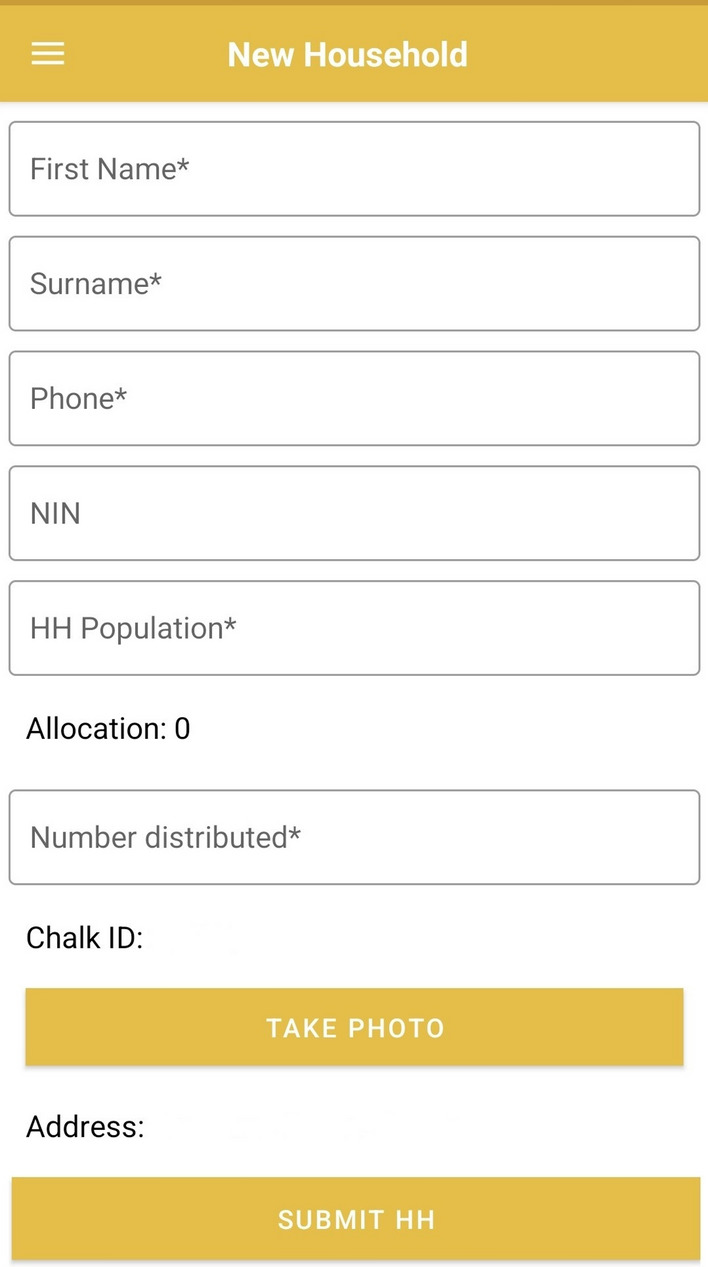
Data Entry Page of EDMIS app

To accommodate places with weak or no internet service, the system's data-gathering module was designed as an Android-based app with online and offline functionality. The electronic data collection form used in the EDMIS app collected data on four elements: the household head's first and last name, phone number, National Identification Number (NIN), and household population (Fig. 1). Furthermore, the app-enabled distribution teams to compute net allocations for each household automatically. There was also a field for entering the number of distributed nets in each household. Unique identification of registered households was accomplished by automatically generating a chalk ID on each household's physical building or house to facilitate distribution supervision.

The EDMIS mobile application provided 5% data verification by parish leaders in each parish. The 5% verification technique involved randomly selecting and re-visiting 5% of all registered households (100%) in a parish to check data quality.

The web-based platform https://edmis.health.go.ug implemented the EDMIS data analysis, visualization, reporting, account creation, and management modules. Access control was ensured through the web-based system, which includes user account creation (for both web and mobile-based services), user account management, and system access limits. This module gave dashboards with insights into the performance of three main indicators: households, population, LLINs, and administrative unit coverage, based on the baseline data obtained during microplanning.

The system was wholly connected with the Central Collaboration Control Information System (CCMIS), allowing for real-time updates and administrative unit management within the EDMIS. The Central Collaboration Management Information System (CCMIS) is a web-based application designed to facilitate collaboration among the campaign's four subcommittees regarding reporting, reconciliation, LLINs and non-LLINs logistics tracking, information sharing, and access.

EDMIS was piloted in March 2020 to simulate system functionality (owning smartphones, testing mobile internet connections, data requirements, and assessing VHT competence and expertise) in a controlled environment. After the software developer resolved all system faults discovered during the pilot test, the system was handed over to the MOH. The system was tested in both urban and rural environments.

Data backup was performed to mitigate data loss and compromise. Other mechanisms included manual household registration forms, autosynchronization, account information extraction, and data entry centres used in locations with insufficient or no network. One thousand nine hundred fifty (1,950) smartphones were distributed across each distribution wave to achieve the digitalization objective. There were also 110 laptops, 1,950 power banks, and 440 wireless routers.

#### Social and behaviour change communication (SBCC)

Key approaches for SBCC were advocacy, mass media, interpersonal communication, information, education, and communication (IEC), and social and community mobilization. The branding of the campaign was dubbed “Under the Net”, which resonated with the campaign objective. National-level advocacy was done to introduce the campaign to national-level stakeholders. This was followed by regional and district-level advocacy meetings. Campaign communications were distributed via mass media, social media, and IEC materials. Community mobilization to increase their participation was done through megaphones and mobile trucks. The call centre at MOH was instrumental in collecting feedback from communities after completing LLIN distribution.

## Results

### Campaign waves

**Fig. 2:**
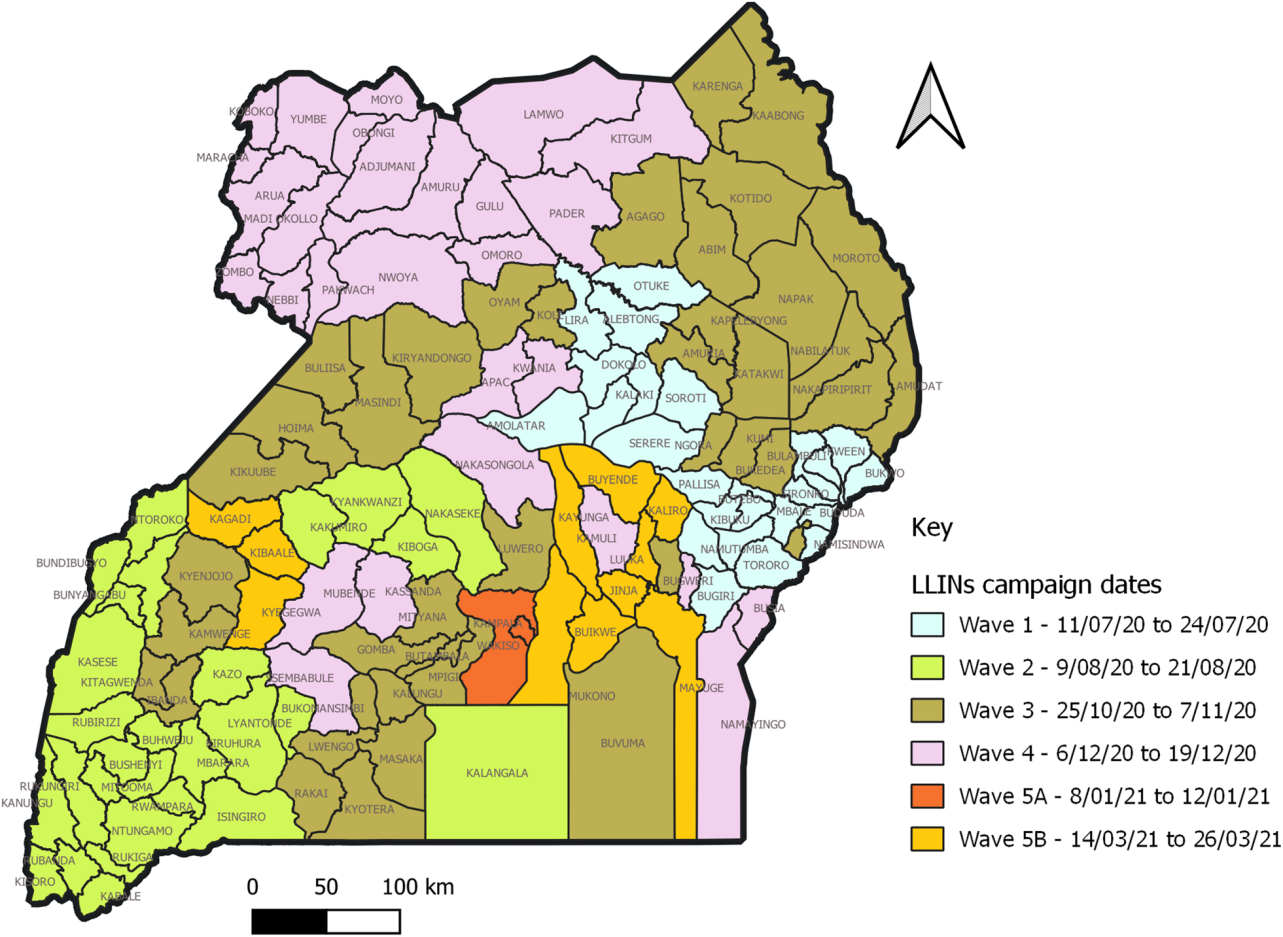
Map of Uganda showing geographical coverage by Wave

Campaign activities were done in five waves to cover the entire country of 136 districts, 2073 sub-counties, 9122 parishes, and 66,742 villages. The breakdown of administrative units reached is shown in Table 1. The waving plan, as shown in Fig. 2, allowed for adequate deployment of resources and managing warehouse space and resources for transportation to sub-counties.

**Table 1:** Duration and cost of in-country distribution of LLINs in the three mass LLIN campaigns

Mass campaign	Campaign dates	Duration (months)	LLINs distributed	In-Country distribution Cost ($)	In-Country distribution Cost/LLIN ($)
2013/2014	May 2013-August 2014	16	21,710,836	19,840,091	0.91
2017/2018	Feb 2017-March 2018	13	26,282,952	22,117,508	0.84
2020/2021	July 2020-March 2021	9	27,800,000	33,657,830	1.21

### Household registration and distribution:

A total of 57,327,395 people (less 0.03% from projection), including refugees, were registered in 11,287,392 households (less 5.9% from projection), with an average of 5.1 persons per household (Table 2). A total of 27,789,044 LLINs were distributed to 11,287,392 households, with 2.5 LLINs distributed per household on average. LLINs were distributed to all registered households. Most LLINs (23.5%), corresponding to 6,543,691 LLINs, were distributed in Wave 5 districts. Moreover, 99.97% (57,327,395 people) of the projected population and 94.1% of the projected households received LLINs during distribution.

**Table 2:** Registered Population, Households, and LLINs distributed

Campaign Wave	Household enumeration				Population enumeration				LLINs distribution			Average number of people per household	Average Number of LLINs distributed per household
	Projected	Registered and Served	Variation (%)	Coverage (%)	Projected	Registered and Served	Variation (%)	Coverage (%)	Allocated	Distributed	Proportion (%)		
1	1,570,733	1,593,085	1.4	101.4	7,468,606	7,586,426	1.6	101.6	4,379,320	4,368,102	99.7	4.8	2.7
2	1,773,865	1,923,341	8.4	108.4	8,638,139	9,658,152	11.8	111.8	5,015,361	4,904,188	97.8	5.0	2.5
3	2,974,614	2,675,335	− 10.1	89.9	13,981,981	13,539,176	− 3.2	96.8	6,848,314	6,365,948	93.0	5.1	2.4
4	2,384,490	2,181,377	− 8.5	91.5	12,464,779	12,290,527	− 1.4	98.6	6,148,800	5,607,115	91.2	5.6	2.6
5	3,293,290	2,914,254	− 11.5	88.5	14,790,762	14,253,115	− 3.6	96.4	7,277,559	6,543,691	89.9	4.9	2.2
Total	11,996,991	11,287,392	− 5.9	94.1	57,344,267	57,327,395	− 0.03	99.97	29,669,355	27,789,044	93.7	5.1	2.5

Of the 28,806,880 LLINs procured, 27,789,044 were given to beneficiaries. Out of 28,805,800 LLINs delivered by NMS to districts, 240,106 LLINs (0.85%) underwent reverse logistics back to the NMS.

### Modifications of the campaign because of COVID-19

The launch of the 2020/2021 campaign was interrupted by the COVID-19 pandemic. The campaign design was modified with the support of the Alliance for Malaria Prevention (AMP) and other malaria partners. The modifications were:

i) Use of Personal Protective Equipment (PPE) such as gloves, masks, aprons, gum boots and hand sanitizers. These were not budgeted initially, but funds from the GF malaria grant savings were available. Due to the global demand surge, it was initially challenging to find PPE items abroad. Due to this, most PPE products, except gloves, were purchased locally. PPE was provided to over 363,568 campaign workers. VHTs were given aprons and gum boots because they were the only ones who required them, while household members received LLINs. District and sub-county supervisors emphasized the importance of wearing PPE, which campaign workers mostly followed.
ii) Utilization of e-platforms for training and briefing meetings. Training at the national and central levels was done using Zoom. Trainers had received instruction in running virtual classes successfully. Meetings and training were conducted online during the initial wave utilizing the Zoom program. This, however, proved insufficiently ineffectual to guarantee that participants learned the skills needed for carrying out activities. This led to a shift toward a mix of physical and virtual meetings and training at all levels. Following the initial training of trainers for the national secretariat and district supervisors, each campaign wave included a single training for sub-county supervisors. Training effectiveness was assessed using pre-and post-tests, which were used to identify trainees who required additional supervision in the field. The number of trainings and meetings was like in previous campaigns [23].
  Physical classes at districts facilitated practical training and improved the evaluation of training efficacy. Furthermore, physical classes were held on the open grounds of sub-county and district offices. This allowed for social distancing between trainers and participants, who could sit at least 1–2 m apart during training sessions. Prior campaigns relied on physical training sessions, which required hiring halls for $ 54.05 per hall at the district and sub-county levels. The 2020/2021 campaign would have spent $107,180 at that price.
iii) Electronic data management systems with the use of smartphones. An app was designed to be used on any Android smartphone. Hard copy registration forms were a backup method in places with poor or no internet access. EDMIS and CCMIS were used to collect data electronically. Following consultation with all partners, the number of data elements gathered at the household level was reduced to a bare minimum of five. Data components like sleeping areas, expectant mothers, and young children were removed. This ensured that little time was spent in each home, reducing the possibility of transmitting and being exposed to COVID-19. DECs collected household data and worked with VHTs to ensure data transfer was completed on time. Previously, VHTs collected household data and distributed LLINs through manual registration forms. Following that, household registration data was entered at the data centre. This information was then shared with NMCD to inform LLIN allocations and dispatch [23].
iv) Simultaneous household registration and distribution of LLINs. Consideration was made to limit contact and movements by the distribution staff and avoid gathering beneficiaries at distribution points, as was done in previous campaigns. Prior campaigns relied on household registration to determine LLIN needs, with distribution occurring at fixed distribution points close to the settlements [15]. The strategy was changed to a single step that combined household registration and ITN delivery door-to-door. This technique was found to have poor accountability for LLINs throughout the first wave of the campaign. In later waves, the method was changed to include household registration in the morning and distribution in the afternoon, both on the same day.
v) The procurement and financial management organization contracted by the MOH made all payments online via mobile money. Payments were made following submitting and verifying payees' names and mobile phone numbers. Cash payments were avoided because they would stimulate payee congregation, increasing the chance of COVID-19 transmission. However, weak network connectivity and capacity concerns slowed the bank's adoption of mobile money payments. There were several examples of late payments and phone numbers registered in names other than of the real payees. Previous campaigns relied on cash payments.
vi) Microplanning. Household registration guided the pre-positioning of LLINs at the district, sub-county, and parish levels before the COVID-19 pandemic. Instead, microplanning data was used during the 2020/2021 campaign to determine LLIN pre-positioning. To provide high accuracy in establishing population numbers and LLIN quantities, this technique required improvement of microplanning, which was previously done at the sub-county level. Teams for microplanning visited villages to verify population and household data.

### Cost and duration of the campaign

The distribution cost for each LLIN was $ 1.21 in the 2020/2021 campaign. This was considerably higher than the cost in the 2013/2014 and 2017/2018 campaigns, which were $ 0.91 and $ 0.84 respectively. The high cost can be attributed to PPE ($ 2.95 m), data entry for household registration and distribution ($ 5.72 m), and the upgrade to EDMIS ($ 225,000).

PPE included 429,254 bottles of alcohol-based hand sanitizers, 60,369 packs of examination gloves, 429,254 cloth masks, 122,644 gum boots, and 122,644 plastic aprons. Using data entry clerks and local guards increased the expense of data entry at the household level. Because pilot testing of EDMIS revealed that most VHTs did not own smartphones, data entry was performed by data clerks who owned smartphones. The data management system was upgraded, which necessitated the hiring of a consultant and the purchase of new hardware.

The 2020/2021 campaign lasted nine months. Despite its late start, the campaign was shorter than the previous ones.

## Discussion

The 2020/2021 LLIN campaign achieved household coverage of 94.1%. The campaign’s objective was thus met despite the difficulties caused by COVID-19-related disruptions. The campaign’s coverage is consistent with a study conducted in 14 districts three months after the 2020/2021 campaign, which found that nearly all (96%) surveyed households owned at least one LLIN [24]. Another study conducted 1–5 months after distribution in 12 districts found that more than 93% of households had at least one LLIN obtained through the 2020/2021 campaign [17]. A similar household coverage of 93.35% was obtained in Benin’s 2020 mass LLIN campaign [25]. On the other hand, the 2020/2021 achieved a higher household coverage than the 2013/2014 (90%) and 2017/2018 (72%) campaigns [26, 27]. The high household coverage in 2020/2021 could be attributed to two factors: first, the door-to-door distribution strategy [17, 28], and second, the high availability of household members at home due to COVID-related lockdowns [17]. Mass campaigns are, therefore, effective in increasing net coverage in the population [29]. However, mass campaigns should be supplemented with other routine distribution methods like schools and health facilities to increase coverage to 100%. The private sector vendors have a role in providing affordable ITNs, especially in urban areas.

Except for wave two districts, the population registered and served was nearly identical to the population projection (0.03%). The population was overestimated by more than 11.8% in wave two districts of the 2020/2021 campaign due to a lack of robust verification of household members during the micro-planning and distribution exercise. The household heads might have overstated the number of household members because it was associated with the number of nets received. Capping the maximum number of nets had limited success in reducing excesses of household members. Studies in other countries document similar occurrences [30].

Social distancing and the use of PPE made a possibility for the LLIN campaign to be carried out in the context of the COVID-19 pandemic. The exercise of net distribution is known to attract crowds of beneficiaries when distribution points are used, which was avoided by carrying out door-to-door household registration and distribution. This demonstrated that malaria prevention activities could be safely implemented in the face of a COVID-19 pandemic. This is even more significant because the pandemic disrupted the population's access to healthcare services because of lockdowns, limited transport means, and fear of contracting COVID-19 from health facilities [12].

Virtual training was successful in cascading training at the national level. Large groups of supervisors were trained and equipped with skills for effectively implementing campaign activities. E-training reduced the costs of hiring venues and the risk of spreading COVID-19. Other education programmes in schools and universities also adopted e-learning to maintain academic calendars [31].

The electronic app was significant in this campaign. It limited the use of manual forms, which would slow the exercise of household registration. Additionally, there was no need for centralized data entry centres like in prior campaigns, which minimized costs and congregation of many people. Data was accessed from dashboards on the progress of the campaign in real-time. Data could be utilized to redistribute LLINs from villages with excess nets to those depleted on the distribution days. Electronic data management has been used in LLIN campaigns in other countries, providing effective implementation of the campaign activities [25, 30]. Adapting EDMIS for other settings and initiatives, such as childhood immunization days, is proposed.

It was more expensive to distribute LLINs during the COVID-19 pandemic due to changes such as campaign staff wearing PPE, upgrading IT equipment, and adding personnel to the distribution team. An increase in personnel for household distribution was required to facilitate door-to-door registration and LLIN delivery. Previous research has found that door-to-door distribution is more expensive than fixed distribution points [32].

However, some challenges were encountered. Simultaneous registration and distribution of nets allowed the distribution team to deliver more nets to households than in the guidelines. By separating registration and distribution after the first wave of the campaign, the challenge of oversupply of LLINs was lessened.

Electronic data management was difficult in areas with poor internet and grid connections. This was mitigated by manual data extraction from phones and the use of charging centres, respectively. The two interventions were, however, time-consuming but valuable. The first wave of distribution experienced a complete failure of the EDMIS app, necessitating the use of paper as a backup. The failures of the app were corrected in the subsequent waves to eliminate any use of paper. The success of apps in such exercises depends heavily on the availability of compatible mobile smartphones and the ability of data entrants to quickly learn how to use the app.

Microplanning data was, in some cases, overinflated. This could have been due to a lack of accurate data in the administrative units [30]. This was common in districts of the first and second wave. This was addressed by comparing data from the previous campaign of 2017/2018. However, the extent of mitigating data inaccuracies was limited by the mass movement of people to villages during the COVID-19 lockdown [33].

## Conclusions

Coverage of LLINs was high. Mass distribution of mosquito nets every three years should be adequately supported as an effective measure for malaria prevention. Registration data was often overstated. Microplanning should be done up to the village level, and validation should be done to ensure accurate figures. Electronic data management using EDMIS was effective in managing the data of the mass campaign. It should be improved by investing early in app design and conducting pilots to troubleshoot any issues.

## Data Availability

The data sets, and reports analysed during the current study are available from the corresponding author on reasonable request.

## Abbreviations

AMF: Against Malaria Foundation
AMP: Alliance for Malaria Prevention
GFATM: Global Fund Against AIDS, Tuberculosis, and Malaria
HBHI: High Burden High Impact
M & E: Monitoring and Evaluation
MOH: Ministry of Health
NCC: National Coordination Committee
NMS: National Medical Stores
PBO: Piperonyl-Butoxide
PPE: Personal Protective Equipment
PMI: President’s Malaria Initiative
SBCC: Social and Behavior Change Communication
UBOS: Uganda Bureau of Statistics
USAID: United States Agency for International Development
VHT: Village Health Team
WHO: World Health Organization
